# Resnet-Driven In Silico Identification of Lead Peptides from the Venom Gland Transcriptome of *Orientothele washanensis* Coupled with Molecular Docking and Dynamics Simulation

**DOI:** 10.3390/ph19091368

**Published:** 2026-08-29

**Authors:** Xin Zeng, Wen-Feng Du, Wen-Hao Yin, Jun-Yao Zhu, Yu-Bin Yang, Wei-Jun Guo, Wen-Liang Li, Hao Gong, Zi-Zhong Yang, Yi Li

**Affiliations:** 1 College of Mathematics and Computer Science, Dali University, Dali 671003, China; xinzeng@dali.edu.cn (X.Z.);; 2 Yunnan Provincial Key Laboratory of Entomological Biopharmaceutical R&D, Dali University, Dali 671000, China; 3 College of Agriculture and Biological Science, Dali University, Dali 671003, China; 4 School of Basic Medical Sciences, Dali University, Dali 671000, China

**Keywords:** *Orientothele washanensis*, lead peptide screening, deep residual network, molecular docking, molecular dynamics simulation

## Abstract

**Background**: *Orientothele washanensis* is a venomous spider with considerable ecological and scientific importance. Its venom, characterized by complex composition and ease of collection, serves as a valuable resource for the discovery of natural peptide drugs. Conventional wet-lab screening methods are limited by rigorous experimental conditions, high resource consumption, and long research cycles, which hinder the efficient identification of functional peptides from the venom gland transcriptome of this spider. **Methods**: To address these technical bottlenecks, this study developed a novel deep learning model named PepPI-DRN for peptide-protein interaction prediction. The model integrated a residual equivariant graph neural network, a residual 1-dimensional convolutional neural network, and a dual-modal attention mechanism by leveraging both sequence and structural features of peptides and proteins. **Results**: Results on an independent test set indicated that PepPI-DRN achieved the competitive or superior performance compared with state-of-the-art methods on multiple key evaluation metrics. Candidate peptides with high interaction probabilities against targets were obtained from the venom gland transcriptome of *Orientothele washanensis*. Furthermore, lead peptides with high binding strength and good structural stability were identified from candidate peptides by molecular docking, and molecular dynamics simulation. **Conclusions**: These results showed that the pipeline with PepPI-DRN, molecular docking and molecular dynamics simulation enabled efficient and reliable identification of lead peptides from the venom gland transcriptome of *Orientothele washanensis*, providing a robust and effective strategy for the discovery and development of natural peptide drugs from the spider venom.

## 1. Introduction

*Orientothele washanensis* (*O. washanensis*) was first discovered in 2022 from Lincang City, Yunnan Province, China, predominantly inhabiting areas at elevations ranging from 1000 to 2000 meters. As a large, venomous, predatory spider, it mainly feeds on insects and plays an important role in pest regulation within the ecosystem [1]. Additionally, due to its easily accessible venom and complex, diverse toxic components, this species has become a key research subject for the discovery of natural peptides. Systematic analysis of its genetic information and toxin composition holds significant potential for advancing applications in both pest control and pharmaceutical development [2,3,4,5,6].

Currently, more than 300,000 sequences have been obtained from the venom gland transcriptome of *O. washanensis*. By integrating the transcriptomic and proteomic data with conventional bioinformatics tools [7,8], researchers have preliminarily uncovered the diversity of its venom molecules and discovered multiple potential active peptides. However, traditional analytical approaches are hampered by long processing times and low throughput, which limits the systematic high-throughput screening of toxic active components. As a result, the therapeutic potential of many toxin-derived peptides remains largely untapped. For example, Zhang et al. [1] utilized transcriptomic and proteomic profiling and uncovered 69 putative toxin sequences from the venom of *Macrothele washanensis*. Notably, 37 of these sequences, exceeding 53% of the total, exhibited a sequence homology below 50% compared with existing database entries. Sequence homology-based methods such as BLAST depended heavily on existing databases and achieved an identification rate of less than 5% for novel peptides lacking known homologous sequences [9]. Molecular docking (e.g., AutoDock Vina) required over two hours for a single peptide-protein docking event [10]. When facing the combination of hundreds of thousands of sequences and multiple target proteins, the computational timeline can exceed one year, making it impractical for high-throughput screening requirements. In light of these challenges, the development of a novel deep learning-driven screening method that balances both “high precision” and “high throughput” characteristics holds significant promise for improving the efficiency of lead peptide screening from the venom gland transcriptome of *O. washanensis*, thereby accelerating the development of peptide-based therapeutics [11,12].

Deep learning has been widely applied across multiple fields with remarkable achievements. For instance, it has enabled efficient screening of lead small molecules by predicting drug-target interactions or binding affinity [13,14,15,16,17]. It facilitated the identification of protein-protein interaction sites, thereby contributing to the elucidation of disease mechanisms and promoting the development of novel therapeutics [18,19,20,21]. It aided in determining the presence of protein-protein interactions, deepening our understanding of cellular functions under both normal physiological and pathological conditions [22,23]. Furthermore, in recent years, deep learning techniques have been increasingly employed in the emerging field of peptide-protein interaction (PepPI) prediction, providing a new research paradigm for lead peptide screening [24,25,26]. Current deep learning-based methods for PepPI prediction can be broadly divided into two categories: docking-based methods and sequence-centric approaches.

In the field of peptide-protein docking, various deep learning-based methods have exhibited good performance. RAPiDock [27] employed a rational and accurate diffusion generative model to achieve high-precision peptide-protein docking at the all-atom level, providing an effective tool for screening lead peptides against pathogenic targets. AlphaFold-Multimer, when combined with forced sampling strategies, improved the quality of peptide-protein docking [28]. Another approach streamlined the AlphaFold2 workflow for generating peptide-protein complexes without requiring multiple sequence alignments of peptide partners, thereby enabling the rapid and accurate peptide-protein docking model [29].

In the field of PepPI prediction, existing approaches have employed convolutional neural networks (CNNs), recurrent neural networks (RNNs), Transformers, and pre-trained models to extract deep sequential features from peptide and protein sequences for predicting their interactions [30]. For example, SWING [31] relied solely on the sequence information to design an interactive language model, utilizing amino acid property variations to construct an interaction grammar with interactive windows, thereby learning the interaction language between peptide and protein. IIDL-PepPI [32] modeled the contextual information of peptides and proteins via a bidirectional attention module and incorporated a progressive transfer learning framework to identify specific binding residues for predicting PepPI. PepBAN [33] first leveraged the protein pre-trained model ESM-2 [34] to represent peptides and proteins, then applied CNN to further extract deep features, followed by a bilinear attention network to integrate their representations, and finally introduced conditional domain adversarial learning to enhance generalization ability across diverse protein targets. DeepPepPI [35] developed a data-driven contextual embedding module and a dual-level auto-correlation search module to capture the rich semantic information from peptides and proteins, respectively, and employed a cross-independent feature integration module to fuse their features, ultimately utilizing a multi-layer perceptron (MLP) to determine the presence of interactions. TPpred-ATMV [36] constructed a latent subspace among multi-view features derived from the therapeutic peptide sequences and proposed an auto-weighted multi-view tensor learning model that leveraged high inter-feature correlations to identify therapeutic peptides. CAMP [37] integrated CNN with self-attention mechanisms to extract diverse features from peptide and protein sequences, simultaneously accomplishing both PepPI prediction and binding sites identification.

Other studies have introduced graph neural networks (GNNs) on the basis of peptide and protein sequences to extract structural features and integrate the sequence and structural information, thereby improving the prediction performance of PepPI. For instance, TPepPro [38] constructed a dual-linear architecture, using CNN and GNN to extract sequence and structural features, respectively, and then fused the two types of features to predict the interactions through a fully connected network. sAMPpred-GAT [39] relied solely on the sequence and structural information of peptides, constructed the peptide structure as a graph, used sequence features as node features, extracted the structural information via a graph attention network (GAT), and finally discriminated whether it was an antimicrobial peptide through a fully connected network.

Based on the analysis of existing research, it reveals three main shortcomings in the current field of lead peptide screening: first, existing deep learning-based models for predicting PepPI do not sufficiently utilize the structural information. Most methods rely solely on the sequence data or employ structural feature extraction techniques that lack spatial equivariance, making it difficult to effectively capture the conformational diversity of biomacromolecules. Moreover, the prediction accuracy of these models generally hovers around 0.85, indicating the considerable room for improvement. Second, the application scenarios of existing models are relatively limited. Most are developed on generic datasets, with no dedicated studies on lead peptides screening for specific species such as *O. washanensis*, and there is a lack of optimization for short peptides and novel peptide structures. Finally, methods such as deep learning-based PepPI prediction, molecular docking, and molecular dynamics simulations [40] are rarely used in an integrated manner, and a multi-angle collaborative optimization strategy for lead peptides screening has yet to be established.

To address the aforementioned challenges, this study proposed a deep residual network architecture named PepPI-DRN for screening candidate peptides from the venom gland transcriptome of *O. washanensis*. Furthermore, we employed molecular docking and molecular dynamics simulation to discover lead peptides in candidate peptides. The major contributions of this work are summarized as follows:

(1) Based on the sequence and structural information of peptides and proteins, this study developed a deep learning model termed PepPI-DRN for PepPI prediction. Compared to existing methods such as TpepPro, PepBAN, DeepPepPI, and CAMP, the model integrated a residual equivariant graph neural network (EGNN), a residual 1D-CNN, and a dual-modal attention fusion mechanism. Results on the independent test set showed that PepPI-DRN achieved performance close to or surpassing state-of-the-art comparative methods across multiple key evaluation metrics. Furthermore, analysis of gradient changes during model training further verified the effectiveness and reliability of PepPI-DRN.

(2) PepPI-DRN was employed to predict the interactions for screening candidate peptides between 30,000 peptide sequences from the venom gland transcriptome of *O. washanensis* and 60 known target proteins. Results showed that PepPI-DRN can effectively identify the potential PepPI, and statistical analyses of these interactions were performed based on the predicted results.

(3) To further discover lead peptides for downstream wet-lab experiments, top-10 pairs of candidate peptide-target complexes with the highest predicted interaction probabilities were selected for the evaluation of binding strength and structural stability by molecular docking and molecular dynamics simulation. Results showed that some peptide-target complexes exhibited reasonable binding conformations and strong binding free energies and displayed favorable structural stability. Collectively, these results indicated that the pipeline, including PepPI-DRN, molecular docking, and molecular dynamics simulation, provided an effective method for high-throughput screening of lead peptides from the venom gland transcriptome of *O. washanensis*.

## 2. Results

### 2.1. Performance Evaluation of PepPI-DRN

To evaluate the performance of PepPI-DRN, we compared it with four state-of-the-art deep learning-based methods on an independent test set: TPepPro [38], TAGPPI [22], PIPR [41], and SCNN. Among them, TAGPPI leveraged deep learning techniques to capture both local and structural features of proteins. PIPR was an end-to-end sequence model based on a Siamese architecture, incorporating a deep residual recurrent convolutional neural network to capture local features and contextual information within protein sequences. In contrast, SCNN was a Siamese convolutional neural network model constructed by removing the GRU structure from PIPR.

Experimental results (Table 1) showed that PepPI-DRN achieved improvements of 3.4%, 9.0%, and 4.0% in ACC, Recall, and F1, respectively, compared to the best values obtained by comparative methods. Meanwhile, slight decreases of 2.8%, 1.2%, and 3.2% were observed in Spec, AUC, and AUPRC. Given that a balanced dataset was used in this study and that the model approached or reached 0.9 on the key metrics such as ACC, F1, and AUC. In addition, PepPI-DRN attained for Precision and 0.778 for Matthews Correlation Coefficient (MCC), two key evaluation metrics for classification tasks. These results indicated that PepPI-DRN exhibited good performance in PepPI prediction.

To further validate the stability and reliability of PepPI-DRN and to eliminate the influence of random factors, we employed a bootstrap resampling approach with 1000 iterations on the test set. In each iteration, samples were drawn with replacement from the test set, and all evaluation metrics were recomputed. 95% confidence intervals (CI) for each metric were then constructed using the 2.5th and 97.5th percentiles of resulting distributions. This approach provides an unbiased estimate of model performance variability without requiring any assumption about the underlying data distribution.

Statistical results (Table 2) showed that PepPI-DRN achieved an AUC of 0.9131 (95% CI: [0.8489, 0.9646]) in PepPI prediction, exhibiting good discriminative ability. Moreover, the relatively narrow width of this confidence interval (width = 0.1157) indicated that the model’s performance was stable under bootstrap resampling. In addition, PepPI-DRN yielded an ACC, Recall, Precision, MCC, and F1 of 0.8839, 0.9643, 0.8308, 0.7780, and 0.8926, respectively, with comparably narrow CI widths of 0.1161, 0.0893, 0.1761, 0.2128, and 0.1164. Collectively, 95% CI and their corresponding widths across all evaluation metrics confirmed that PepPI-DRN achieved well-balanced performance and that its predictions were statistically significant.

### 2.2. Ablation Experiment

#### 2.2.1. Selection of Structural Feature Extraction Architecture

Structural features of peptides and proteins are among the key factors influencing the predictive performance of deep learning-based PepPI. To investigate this, we conducted the comparative experiments on the network architectures designed to extract structural features of peptides and proteins. The architectures under investigation included six types of GNN models: Graph Convolutional Neural Network (GCN), GAT, Graph Transformer, GraphSAGE, EGNN, and Residual EGNN. 

**Figure 1:**
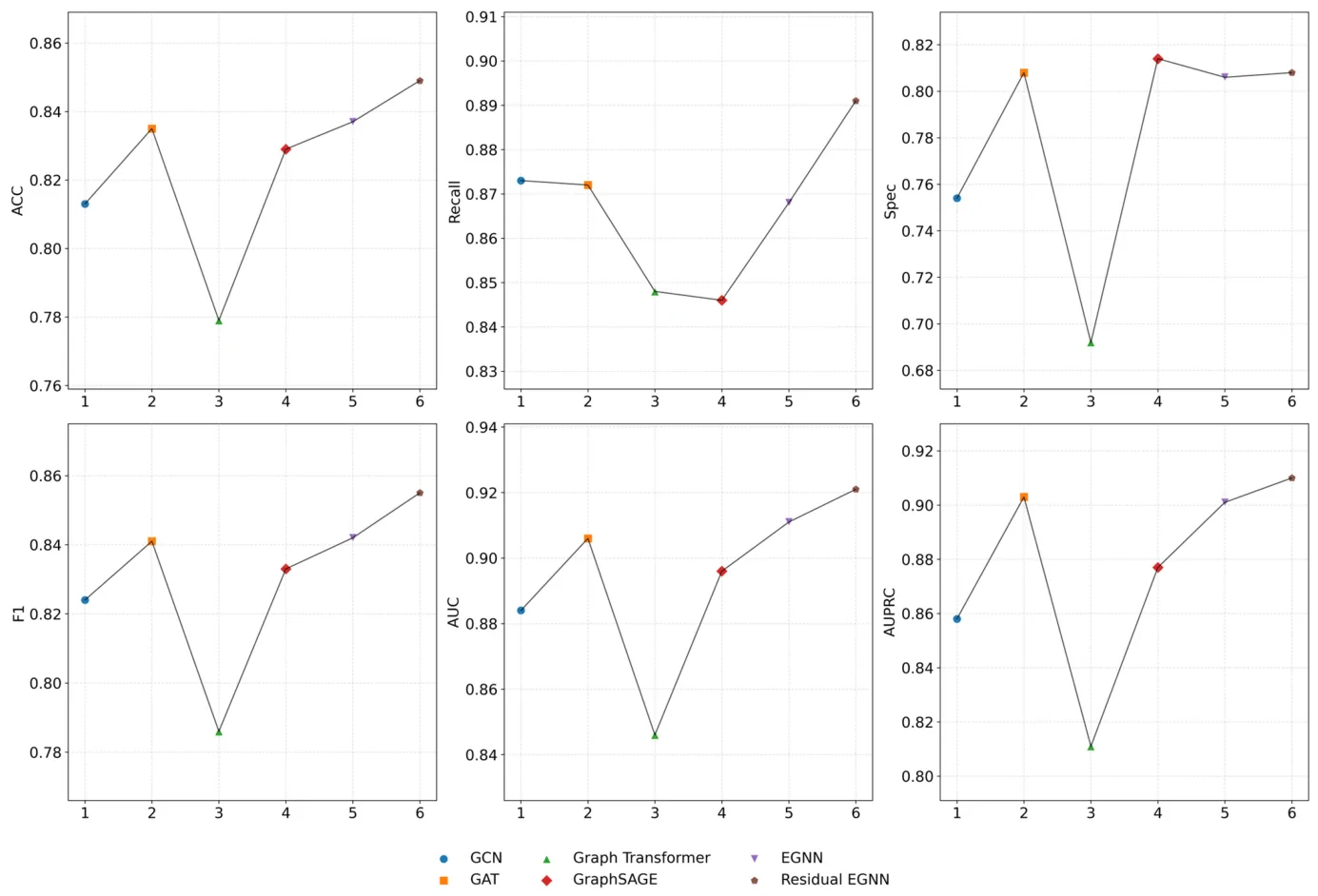
Performance comparison of different structural feature extraction architectures. Residual equivariant graph neural network achieves improvements over optimal results obtained by other networks, with gains of 1.4%, 2.1%, 1.5%, 1.1%, and 0.8% in ACC, Recall, F1, AUC, and AUPRC, respectively.

Experimental results (Figure 1) showed that, aside from slight fluctuations in the performance of Graph Transformer across various indicators, the other five structural feature extraction networks achieved excellent performance, with several metrics approaching or exceeding 0.9. Notably, when employing Residual EGNN, the model achieved improvements over optimal results obtained by other networks, with gains of 1.4%, 2.1%, 1.5%, 1.1%, and 0.8% in ACC, Recall, F1, AUC, and AUPRC, respectively. This performance was only marginally lower than the best value in the Spec metric (a decrease of 0.7%). Based on its better performance, we ultimately chose Residual EGNN as the structural feature extraction architecture for the PepPI prediction model.

#### 2.2.2. Selection of the Number of Layers in Residual EGNN

Residual EGNN was employed to extract structural features of peptides and proteins, and the selection of network depth was critical: too few layers were insufficient to capture deep structural features, whereas too many layers tended to cause feature over-smoothing. To investigate the influence of network depth on model predictive performance, we performed ablation experiments with commonly used settings of 2, 3, 4, and 5 layers while keeping all hyperparameters, including learning rate, optimizer, batch size, and number of training epochs, identical across experiments. Experimental results (Table 3) showed that when the number of layers was increased from 2 to 3, all evaluation metrics improved substantially. From 3 to 4 layers, the metrics declined to varying degrees. And at 5 layers, the performance showed negligible difference compared with 4 layers but remained inferior to that of 3 layers. Collectively, these results indicated that residual EGNN achieved superior performance when configured with 3 layers.

#### 2.2.3. Selection of Sequence Feature Extraction Architecture

After determining the structural feature extraction architecture, we further conducted comparative experiments on sequence feature extraction architectures to select network structures suitable for modeling peptide and protein sequences. These experiments were based on the selected structural feature extraction network, using the sequence information of peptides and proteins as inputs. 1D-CNN and Residual 1D-CNN were employed to extract sequence features, and the performance of multiple model combinations was compared and analyzed.

**Figure 2:**
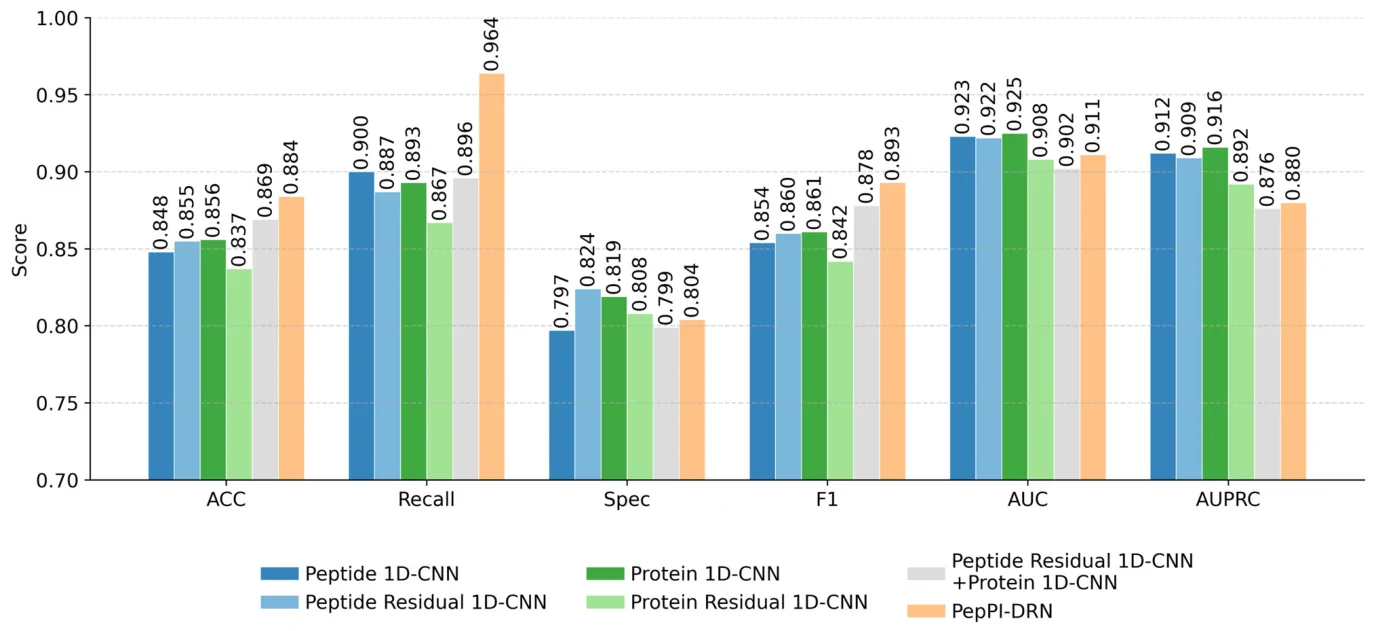
Performance comparison of different sequence feature extraction architectures. Results show that PepPI-DRN achieves improvements of 1.7% and 1.7% in ACC and F1, respectively, compared with the optimal values obtained by other combinations, despite a slight 1.5% decrease in AUC.

Experimental results (Figure 2) indicated that when only peptide sequence features were introduced, Residual 1D-CNN performed similarly to or better than 1D-CNN in key metrics such as ACC, F1, and AUC. When only protein sequence features were introduced, 1D-CNN outperformed Residual 1D-CNN across all evaluation metrics. Although each network exhibited its own advantages in modeling tasks involving only peptide or only protein sequences, we found that in experiments combining both peptide and protein sequences, using a unified architecture (either Residual 1D-CNN or 1D-CNN) was less effective than employing both architectures simultaneously (i.e., PepPI-DRN). Further comparison showed that PepPI-DRN achieved improvements of 1.7% and 1.7% in ACC and F1, respectively, compared to the optimal values obtained by other combinations, despite a slight 1.5% decrease in AUC. Overall, adopting a dual Residual 1D-CNN network to concurrently extract sequence features of peptides and proteins contributed to enhancing the overall performance of PepPI prediction.

#### 2.2.4. Dual-Modal Feature Fusion Strategy Based on Attention Mechanism

**Figure 3:**
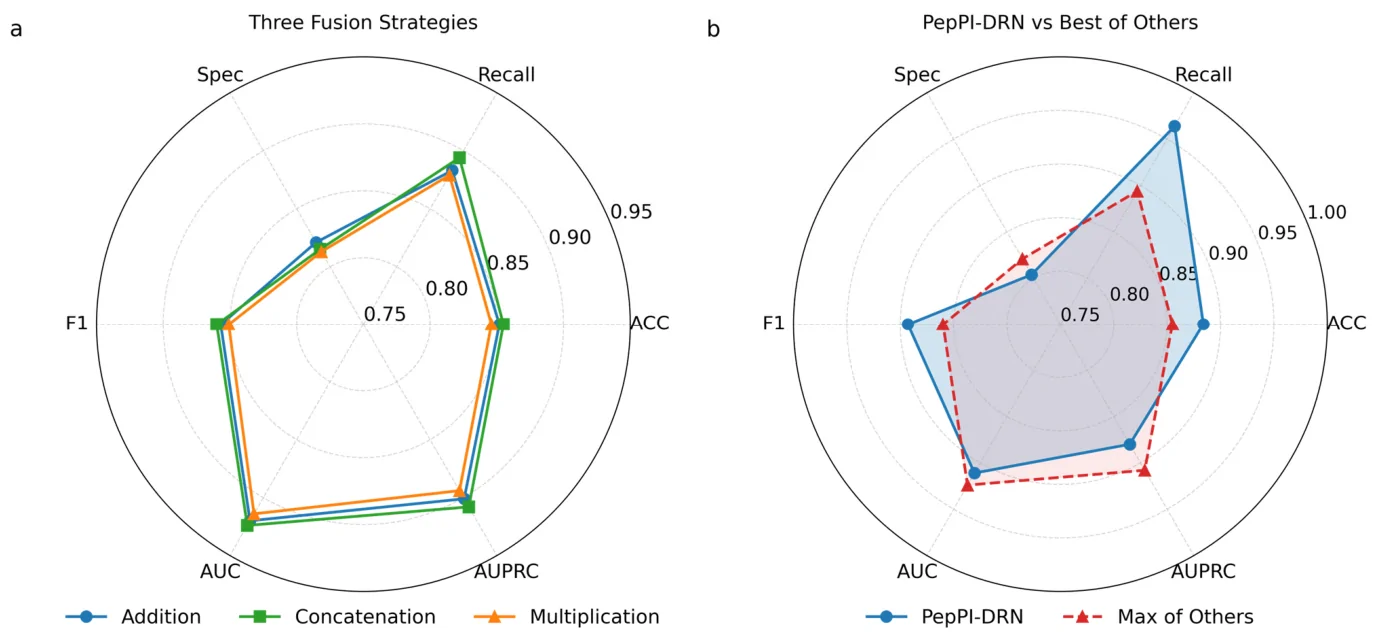
Performance comparison of different feature fusion strategies. (**a**) Results show that three commonly used fusion strategies perform similarly across all evaluation metrics, exhibiting the relatively stable performance. (**b**) The attention-based dual-modal feature fusion strategy (PepPI-DRN) outperforms the best of commonly used approaches by 3.4% in ACC and 3.8% in F1 for the key classification evaluation metrics, despite a slight decrease of 1.4% in AUC.

In the representation learning of peptides and proteins, effectively integrating sequence and structural features enables the acquisition of more comprehensive molecular characteristics, thereby laying the foundation for improving the performance of PepPI prediction. To select the optimal feature fusion strategy, we experimentally compared the attention-based dual-modal feature fusion strategy proposed in this paper with three commonly used fusion strategies: Addition, Concatenation, and Multiplication. Experimental results (Figure 3) showed that three commonly used fusion strategies performed similarly across all evaluation metrics, exhibiting the relatively stable performance. In contrast, an attention-based dual-modal fusion strategy achieved results comparable to or superior to conventional methods in most metrics. Notably, under-balanced datasets, this strategy outperformed the best of commonly used approaches by 3.4% in ACC and 3.8% in F1 for the key classification evaluation metrics, despite a slight decrease of 1.4% in AUC. Overall, the attention-based dual-modal feature fusion strategy made a positive contribution to the model performance improvement.

### 2.3. Interpretability Analysis of PepPI-DRN

**Figure 4:**
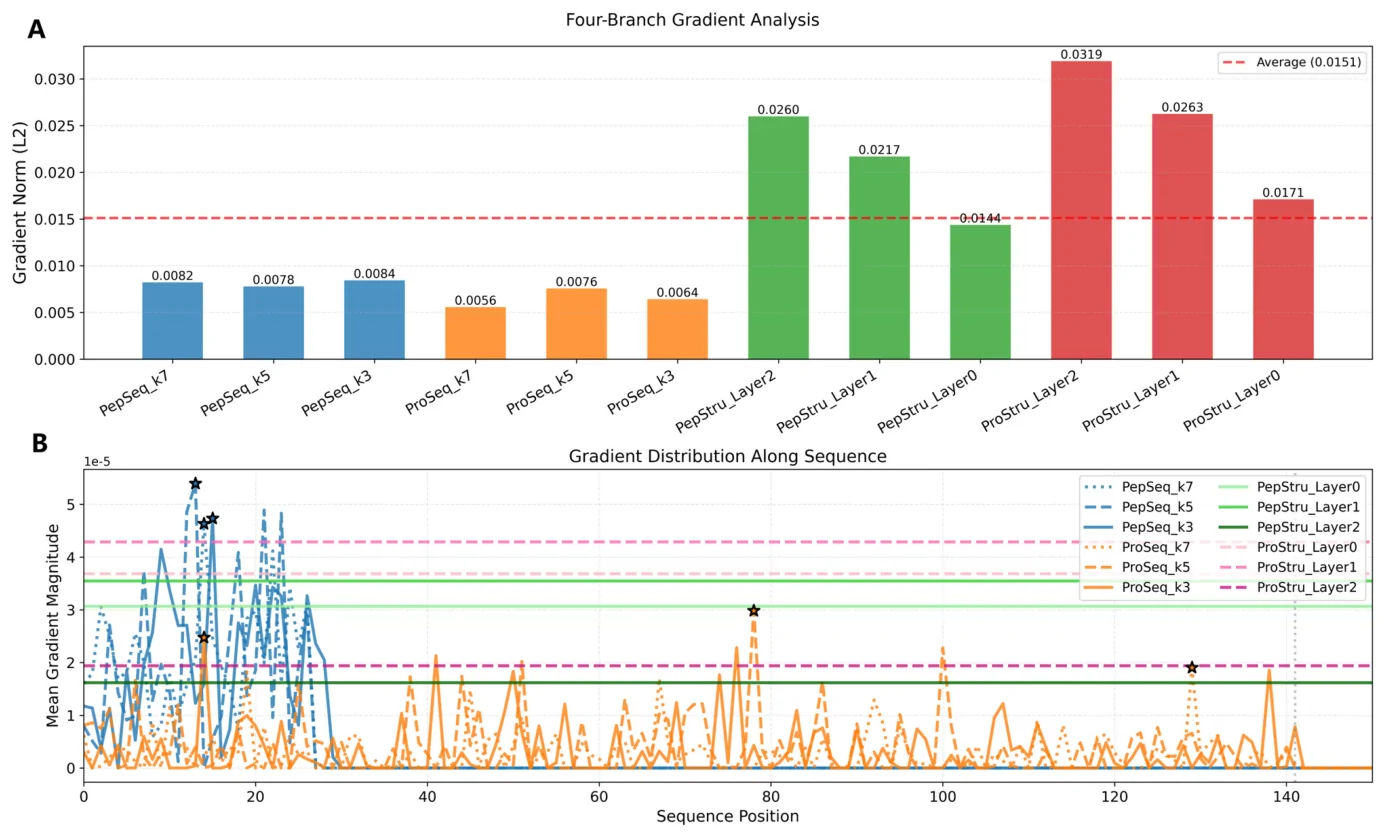
Interpretability analysis of PepPI-DRN. (**A**) The gradient norms of EGNN layers (green, red) are higher than those of CNN layers (blue, orange), indicating that the model relies more on three-dimensional spatial features encoded by graph structure during prediction. Meanwhile, at corresponding layers for structural feature extraction, the gradient norms of proteins are higher than those of peptides, suggesting that protein structural features exert a more critical influence on the model’s structural-based decision-making. (**B**) Both peptides and proteins show obvious gradient peaks at several key amino acid residue positions, and the mean node gradients of GNN layers are overall lower than peak values of CNN layers, demonstrating that the sequence importance of individual key residues exceeds the average contribution of global structural features in this sample.

To elucidate the rationality of the model’s decision-making mechanism, this study employed Gradient-weighted Class Activation Mapping (Grad-CAM) to perform an interpretable analysis of PepPI-DRN from a gradient perspective. Specifically, we calculated the gradients of layer-wise outputs within the network architecture, quantified gradient norms and distribution patterns, and identified the contributions and key functional regions of peptides and proteins in PepPI prediction. Using a pair of positive samples (protein P00766 and peptide 7qit_E) as a case study, we compared and analyzed their gradient L2 norms (Figure 4A) and sequence-position gradient distributions (Figure 4B) between CNN layers and EGNN layers.

A comparison of layer-wise gradient L2 norms revealed that the gradient norms of EGNN layers (green, red) were consistently higher than those of CNN layers (blue, orange) for both peptides and proteins. This result indicated that both sequence and structural information were critical for PepPI prediction, and the model relied more heavily on three-dimensional spatial features encoded by graph structure. Notably, gradient norms of EGNN layers increased continuously from the first to the third layer, indicating progressively refined structural feature extraction. In contrast, CNN layers maintained relatively stable gradient norms when processing peptide and protein sequences, yielding smoother sequence features. Moreover, at equivalent structural feature extraction layers, proteins exhibited higher gradient norms than peptides, suggesting that protein structural features exerted a stronger influence on the model’s structure-based decision-making.

Analysis of residue-wise gradient distributions in the sample showed that the peptide sequence displayed prominent gradient peaks (blue stars) at positions 15, 17, and 18, indicating that these residues contributed crucially to the prediction. The protein sequence exhibited distinct gradient peaks (orange stars) at positions 17, 78, and 129, which may correspond to key binding regions. The mean node gradients of EGNN layers were consistently lower than peak gradients of CNN layers, suggesting that the sequence-level importance of individual key residues surpassed the average contribution of global structural features in this sample. This implied that PepPI was likely dominated by these key residues, whereas other sequence positions and structural regions made relatively balanced contributions.

### 2.4. Screening Candidate Peptides from the Venom Gland Transcriptome of O. washanensis

#### 2.4.1. Candidate Peptides Screening Based on PepPI-DRN

The ultimate goal of developing PepPI-DRN is to screen candidate peptides from the venom gland transcriptome of *O. washanensis*. To achieve this, we first randomly selected 10 target proteins from each of 7 categories of FDA-approved, clinically tested, and reported human target proteins (totaling 859 proteins) [42], including Family A G protein-coupled receptors, ion channels, enzymes, proteases, kinases, transporters, and other enzymes. For categories containing fewer than 10 proteins, all available proteins were included, yielding a final set of 60 target proteins. Each peptide in the dedicated dataset was then paired with 60 selected target proteins to construct peptide-protein pairs. Finally, PepPI-DRN was applied to predict their interactions.

**Figure 5:**
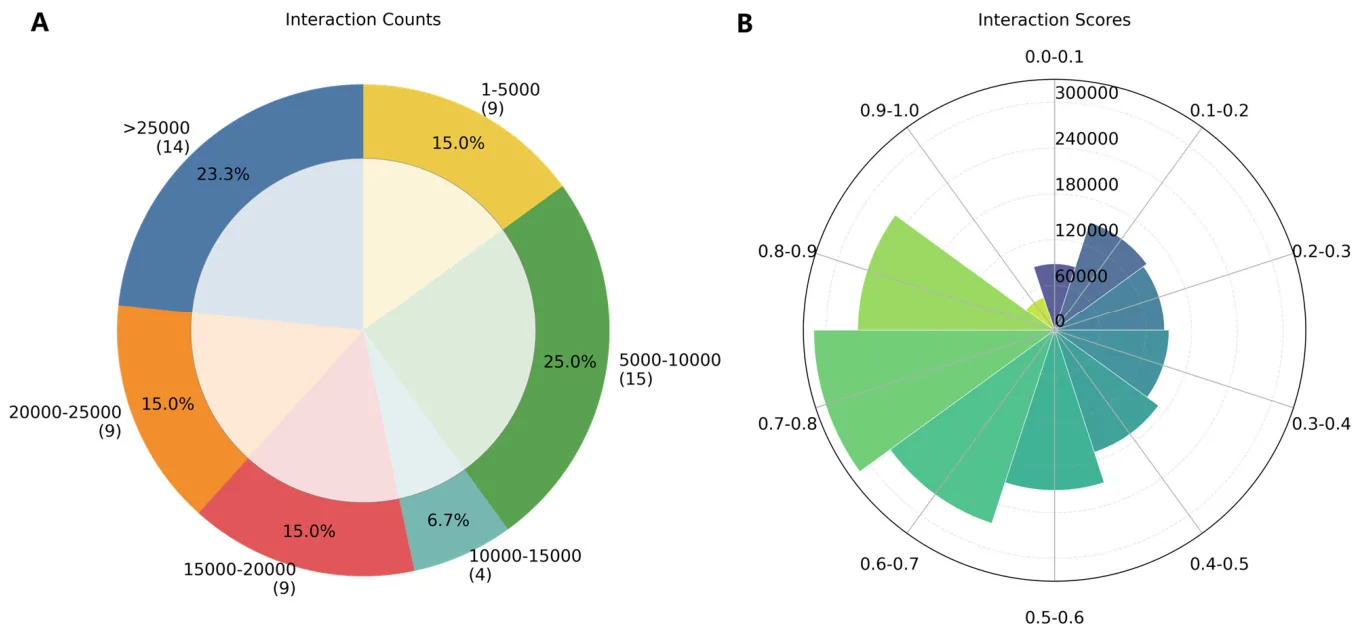
Screening results of candidate peptides based on PepPI-DRN. The interaction profiles are analyzed from two dimensions: (**A**) number of interacting peptides per target and (**B**) interaction probability of each peptide-protein pair.

Experimental results displayed favorable interaction potential between target proteins and sampled peptides. We analyzed the interaction profiles from two dimensions: number of interacting peptides per target and interaction probability of each peptide-protein pair. As shown in Figure 5A, 23.3% of target proteins interacted with more than 25,000 peptides, 15% interacted with 20,000–25,000 peptides; 15% with 15,000–20,000 peptides; 6.7% with 10,000–15,000 peptides; 25% with 5000–10,000 peptides; and 15% with 1–5000 peptides. Regarding interaction probability (Figure 5B), 46,350 peptide-protein pairs exhibited a probability >0.90, 258,617 pairs ranged from 0.80 to 0.90, 316,292 pairs from 0.70 to 0.80, 266,912 pairs from 0.60 to 0.70, 210,543 pairs from 0.50 to 0.60, and 701,286 pairs were below 0.50. Collectively, these findings indicated that the venom gland transcriptome of *O. washanensis* encoded a diverse repertoire of candidate peptides, providing a valuable basis for the development of peptide-based drugs.

#### 2.4.2. Analysis of Binding Strength of Candidate Peptide-Protein Pairs Based on Molecular Docking

**Figure 6:**
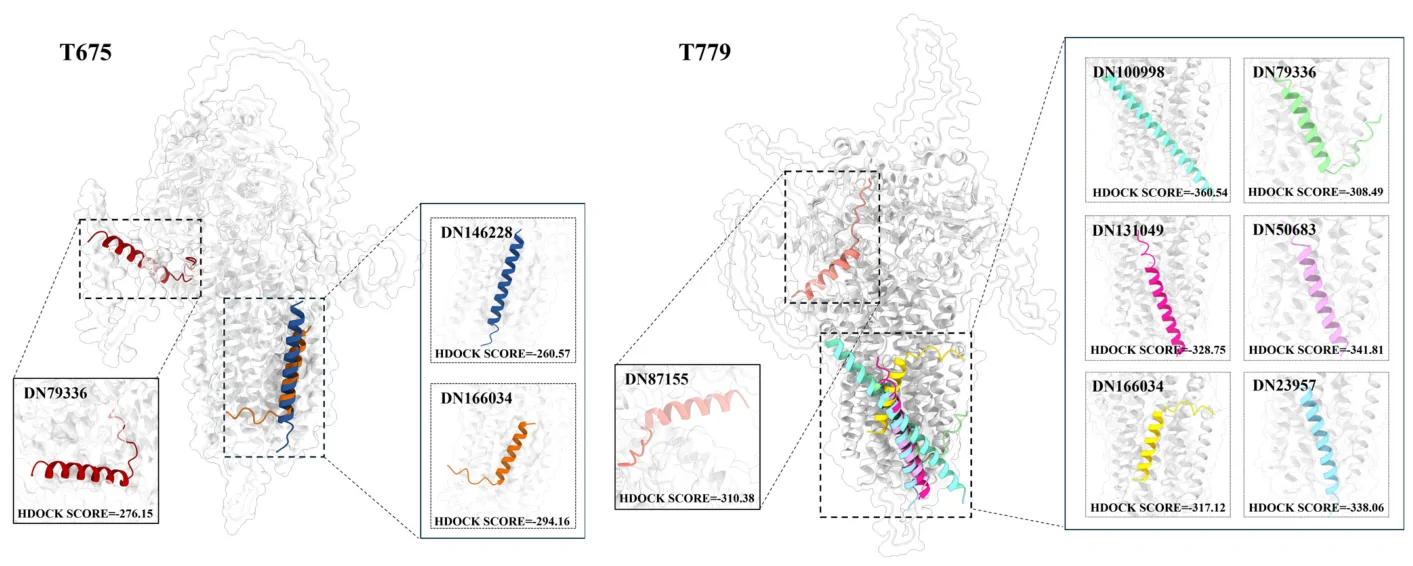
Binding conformations and docking scores of peptide-protein pairs. All predicted peptide-protein pairs display rational geometric conformations and robust binding effects.

To further analyze the binding effects of candidate peptide-protein pairs identified by PepPI-DRN from the venom gland transcriptome of *O. washanensis*, we employed the protein-protein docking tool HDOCK [43] to perform blind molecular docking on the top-10 peptide-protein pairs with the highest predicted interaction probabilities (Table 4). Analysis of docking conformations and docking scores (Figure 6) revealed that all selected peptides could effectively dock with their target proteins, forming reasonable binding poses without obvious steric hindrance or energetic conflicts. The docking scores of T675 with peptides DN79336_c0_g1, DN146228_c0_g1, and DN166034_c0_g1 were −276.15, −260.57, and −294.16, respectively. For protein T779, the docking scores with peptides DN131049_c0_g1, DN87155_c0_g1, DN23957_c1_g2, DN100998_c0_g1, DN50683_c0_g1, DN166034_c0_g1, and DN79336_c0_g1 were −328.75, −310.38, −338.06, −360.54, −341.81, −317.12, and −308.49, respectively. These uniformly low docking scores (lower values indicate better binding propensity, with docking scores generally below −200 considered.) indicated robust binding effects between candidate peptides and their target proteins, supporting the formation of stable peptide-protein complexes.

#### 2.4.3. Structural Stability Analysis of Candidate Peptide-Protein Complexes Based on Molecular Dynamics Simulation for Screening Lead Peptides

To further confirm the structural stability of candidate peptide-protein complexes, we employed molecular dynamics simulation to systematically assess their stability from three key perspectives: root mean square deviation (RMSD), free energy landscapes, and amino acid residue energy contributions.

**Figure 7:**
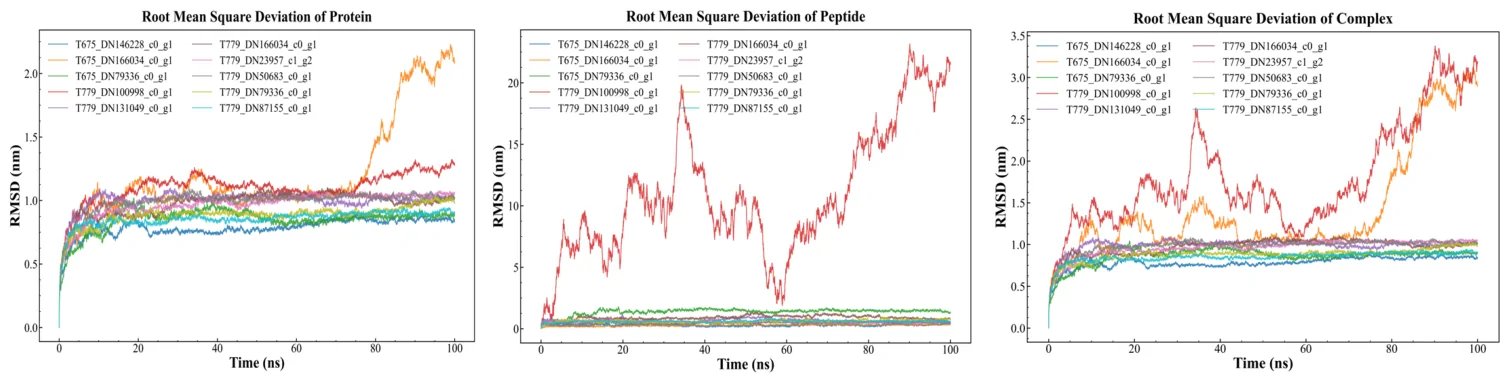
Root mean square deviation analysis of proteins, peptides, and complexes based on molecular dynamics simulation.

**Figure 8:**
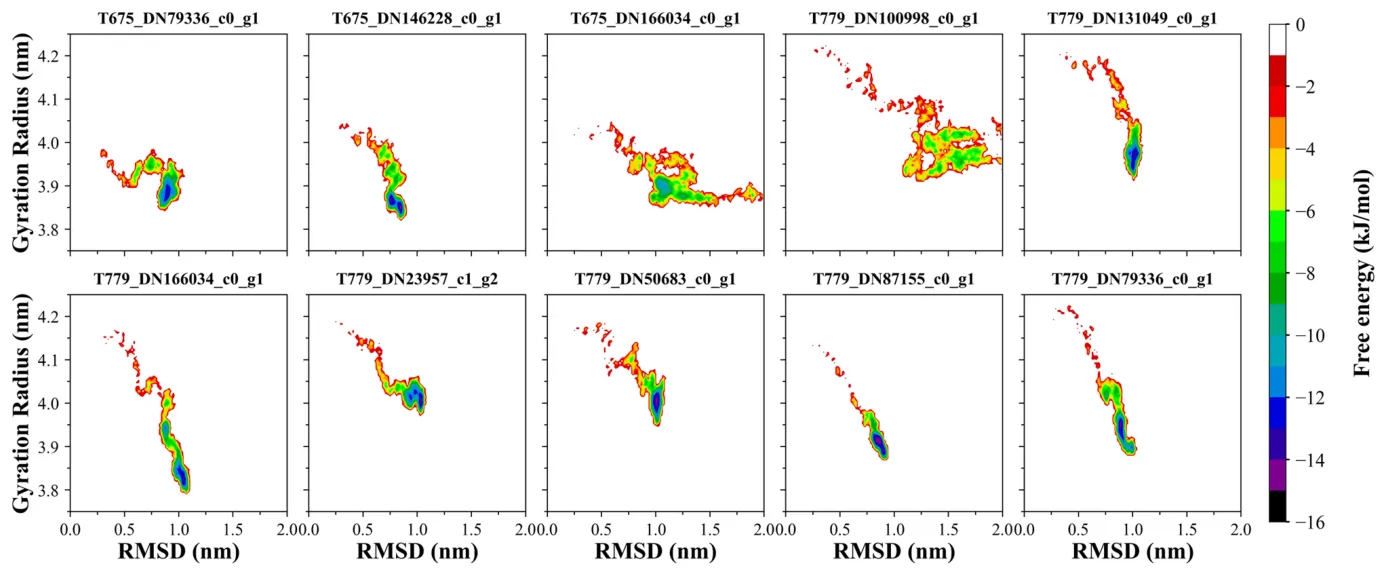
Free energy landscape analysis of candidate peptide-protein complexes based on molecular dynamics simulation.

**Figure 9:**
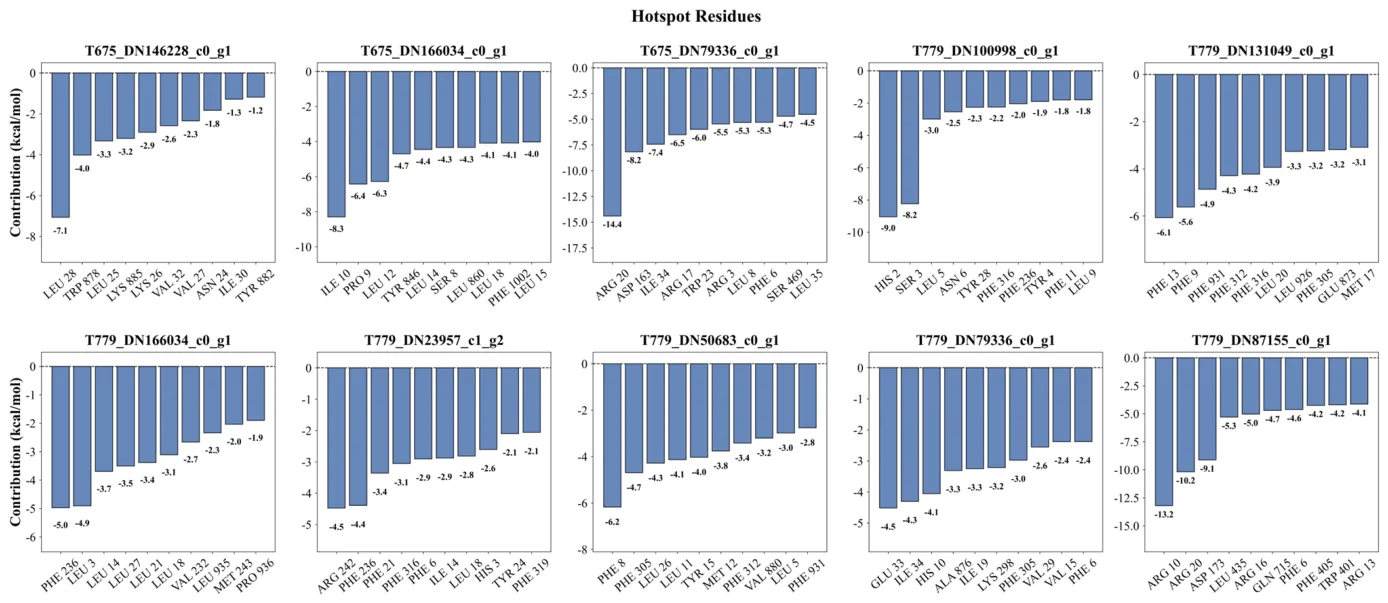
Hotspot residues analysis for peptide-protein binding.

(1) Root mean square deviation analysis of proteins, peptides, and complexes. Over the 100 ns simulation period, most systems exhibited excellent dynamic stability. Following solvation and local conformational relaxation during the initial 10–20 ns, these systems rapidly attained a stable thermodynamic equilibrium (Figure 7). At the protein level, the backbone RMSD converged stably within the range of 0.75–1.1 nm. For the macromolecular systems, this range represented reasonable dynamic fluctuations, indicating that the three-dimensional folded structure of protein was well preserved without obvious conformational disruption. At the peptide level, RMSD curves maintained low magnitudes (mostly below 1.0 nm) with negligible fluctuations, demonstrating that peptides were tightly anchored within the protein binding pocket and formed stable non-covalent interaction networks with surrounding amino acid residues. These results further supported the rationality of initial docking conformations and the reliability of favorable docking scores. System T779_DN100998_c0_g1 (red curve) displayed typical characteristics of binding failure. Although the structure of T779 remained relatively stable, the RMSD of the corresponding peptide rose abruptly at the early stage of simulation and then increased irregularly, reaching values above 20 nm. Such an abnormally high RMSD indicated that the peptide completely dissociated from the protein binding pocket and diffused into the bulk solvent. This observation suggested that even though molecular docking could generate conformations with acceptable scores under the static conditions, the binding force between this peptide and T779 was insufficient to overcome the solvation effects and steric hindrance when an explicit solvent model and dynamic motions were considered. Significant repulsive interactions existed between two molecules, preventing stable complex formation. In contrast, the T675_DN166034_c0_g1 (orange curve) system exhibited remarkable noncanonical stable behavior. In this system, the peptide RMSD remained at an extremely low level (< 1.0 nm) throughout the entire 100 ns simulation, indicating that the peptide did not dissociate or undergo large-scale displacement and remained stably bound to the protein surface. However, in sharp contrast, the RMSD of T675 remained steady during first 75 ns but underwent a pronounced secondary increase after 75 ns, quickly rising above 2.0 nm. This dynamic profile typically indicated a peptide-induced protein allosteric effect, in which sustained intramolecular tension following peptide binding triggered a substantial conformational transition in T675.

(2) Free energy landscape analysis of peptide-protein complexes. During 100 ns conformational sampling, free energy landscapes of most systems displayed highly concentrated funnel-shaped characteristics (Figure 8). These systems rapidly traversed the initial energy barrier and converged into a single deep energy basin. Within this low-energy core region, fluctuations in both RMSD and radius of gyration (Rg) were strictly constrained to an extremely narrow range. Such thermodynamics characteristic strongly indicated that these peptides could form highly specific and rigid interaction networks with proteins, resulting in stable complex structures that resisted dissociation or substantial conformational relaxation. In sharp contrast to the above stable systems, the T779_DN100998_c0_g1 system exhibited an extremely fragmented free energy distribution. It wandered randomly across a broad conformational space defined by RMSD and Rg, with no concentrated low-energy basins observed in the landscape. This extensively dispersed metastable energy distribution pattern provided definitive thermodynamic evidence that the peptide failed to form an effective binding interface with the protein through simulation. Peptide dissociation removed structural constraints on the overall system, making it impossible to maintain a stable complex conformation.

(3) Hotspot residues analysis for peptide-protein binding. By integrating residue decomposition analysis with free energy calculations, hotspot residues in peptide sequences that determine binding specificity were identified at a microscopic level. Analysis (Figure 9) showed that peptides mainly formed high-intensity anchoring with proteins through the following two mechanisms:

(a) Strong electrostatic anchoring and salt bridge formation: In the systems dominated by electrostatic interactions, positively charged arginine (ARG) played a central role. For instance, in the T675_DN79336 system, a single ARG20 residue contributed a binding energy of −14.4 kcal/mol, while in the T779_DN87155 system, ARG10 contributed a binding energy of −13.3 kcal/mol. This type of amino acid possessed long side chains and highly polar guanidine groups, which could penetrate the binding pocket of transmembrane proteins and form stable salt bridges or multiple hydrogen bonds with the acidic residue network inside the protein, thereby firmly locking the ligand conformation.

(b) Hydrophobic interactions and aromatic stacking networks: In some systems (e.g., T675_DN166034 and T779_DN131049), top energy-contributing residues were mainly highly hydrophobic aliphatic amino acids (ILE 10, PRO 9, LEU 12) or aromatic amino acids (PHE 13, PHE 9, PHE 931). These hydrophobic residues tightly adhered to the hydrophobic inner wall of protein via van der Waals forces or formed extensive and stable hydrophobic interaction interfaces through π-π stacking effects. Especially in the T675_DN166034 system, the strong wedging effect of the peptide’s hydrophobic side chains may serve as the core structural basis for inducing conformational changes in T675.

## 3. Materials and Methods

### 3.1. Datasets

In this study, we employed the benchmark dataset consistent with that of TPepPro [38]. All peptide-protein complexes in this dataset were obtained from version 2.3 of the Propedia database [44]. The database comprised a total of 49,300 complexes. After a series of preprocessing procedures, including protein sequence and structural consistency filtering, 19,187 high-quality samples were retained. Among them, 9594 peptide-protein complexes were used as positive samples, and 9593 negative samples were constructed by randomly pairing peptides and proteins from the dataset while excluding those already present in the positive set. Furthermore, we randomly selected 56 positive samples from the benchmark dataset and generated 56 negative samples via random peptide-protein pairing that were not present in the standard dataset, forming an independent test set of 112 samples for model performance evaluation.

The venom gland transcriptome of *O. washanensis* contains 301,013 peptide sequences. To preliminarily identify lead peptides with strong binding strength and stability against given pathogenic targets. We randomly selected 30,000 non-redundant sequences and predicted their tertiary structures using ColabFold (v1.5.5) [45], a tool known for its high prediction accuracy and fast computational speed, thereby constructing a dedicated dataset. As this study represented a preliminary exploration of the medicinal potential of the venom gland transcriptome of *O. washanensis*, and given that all peptide sequences were relatively short (no sequence exceeds 50 amino acid residues), sequence similarity was not evaluated.

### 3.2. Data Representation

#### 3.2.1. Sequence Representation of Peptide and Protein

The sequence representation pipeline adopted in this study was detailed as follows: First, FASTA sequences were parsed using BioPython (v1.85). Sequences containing artificially modified amino acids were filtered out, and non-standard amino acids were replaced with the universal placeholder “X”. Second, irrelevant characters including spaces, line breaks, and annotation information were removed to guarantee that each sequence was a continuous single-letter amino acid string. To satisfy the fixed input dimension requirements of 1D-CNN and improve the efficiency of batch training, we fixed the sequence lengths of proteins and peptides at 1200 and 50, respectively. Sequences longer than the threshold were truncated at the N-terminus, whereas shorter sequences were zero-padded at the C-terminus. Finally, label encoding was applied to achieve a one-to-one mapping between integers and amino acid characters.

#### 3.2.2. Structural Representation of Peptides and Proteins

(1) Construction of graph. Each $C_{\alpha }$ atom in the amino acid residues was treated as a node in the graph. The coordinates of these $C_{\alpha }$ atoms were extracted from tertiary structure files of peptides and proteins. Pairwise, the Euclidean distances between $C_{\alpha }$ were then calculated to construct a distance matrix. To further convert this matrix into an adjacency matrix for graph representation, a commonly used distance threshold of 8 Å was applied: an edge was defined to exist if the distance was below this threshold, with the corresponding entry in the adjacency matrix set to 1. Otherwise, no edge existed, and the entry was set to 0.

(2) Edge features. The edge feature was a 4-dimensional vector, comprising the coordinate differences between two $C_{\alpha }$ atoms along x-, y-, and z-axes, as well as their Euclidean distance.

(3) Node features. The node features within peptide and protein graphs were generated by inputting the corresponding peptide and protein sequences into ESM-2, which output 1280-dimensional feature vectors.

(4) Node coordinates. The node coordinates correspond to $C_{\alpha }$ atom were obtained directly from the tertiary structure file of the protein via BioPython.

### 3.3. Proposed Model

**Figure 10:**
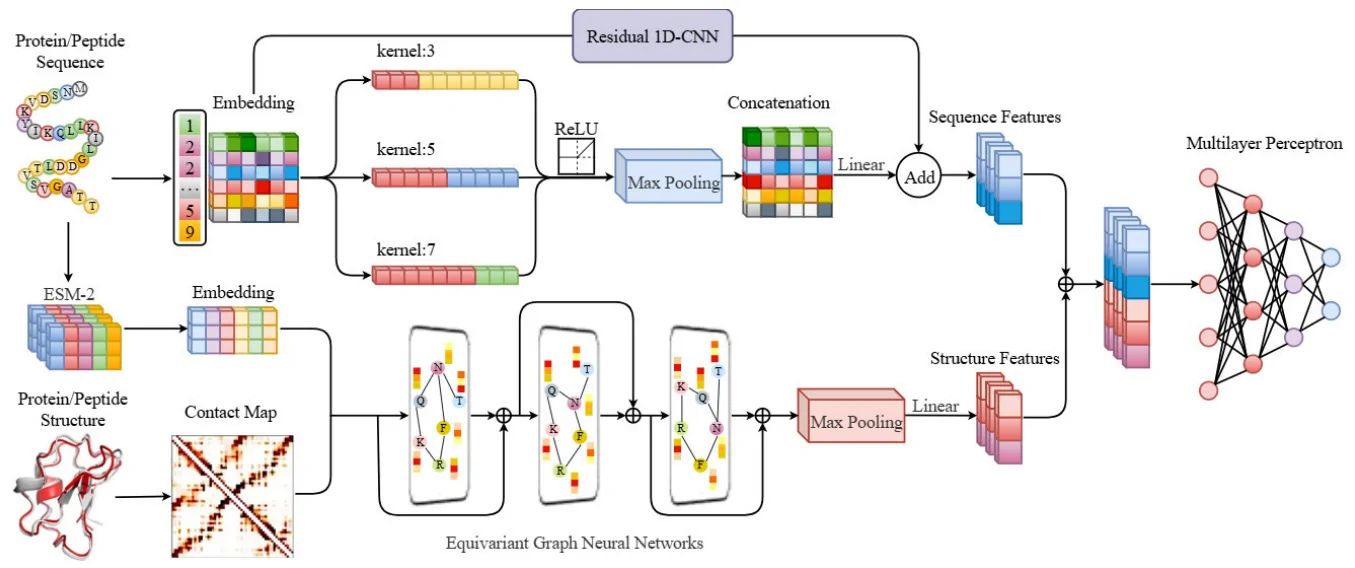
Architecture of PepPI-DRN. Based on the sequence and structural information of peptides and proteins, a residual 1D convolutional neural network and a residual equivariant graph neural network are first employed to extract their sequence and structural features, respectively. Subsequently, these features are internally fused using a dual-modal attention mechanism. Finally, the fused features are externally concatenated and fed into a multilayer perceptron to predict peptide-protein interactions.

In this study, we proposed a novel PepPI prediction architecture, named PepPI-DRN (Figure 10), which was built upon deep residual networks. This architecture extracted sequence and structural features by a residual 1D-CNN and a residual EGNN from peptides and proteins, respectively. A dual-modal attention mechanism was further introduced to effectively fuse the learned features, followed by PepPI prediction via an MLP. Overall, PepPI-DRN comprised four core modules: a sequence feature extraction module, a structural feature extraction module, a feature fusion module, and a PepPI prediction module.

#### 3.3.1. Sequence Feature Extraction Module

The input to the sequence feature extraction module consisted of label encoding of peptide and protein sequences, with a tensor shape of [B, L], where B was the batch size and L was the sequence length. Label-encoded sequences were fed into an embedding layer, which mapped discrete integer tokens to continuous dense vectors, generating an output tensor of shape [B, L, 128].

To extract the discriminative sequence features for peptides and proteins, we designed a multi-scale network based on 1D-CNN to capture contextual information across varying sequence lengths. The network followed a two-stage pipeline: multi-scale convolution and residual fusion. In the multi-scale convolution stage, three parallel convolution branches with kernel sizes of 3, 5, and 7 were adopted to extract short-range, medium-range, and long-range sequential dependencies, respectively. Each branch accepted an input tensor of shape [B, L, 128] and generated a 128-channel output feature vector with a stride of 1, and adaptive padding automatically matched to the corresponding kernel size. The convolutional outputs were activated by ReLU and subjected to global max pooling. Three resulting feature vectors were then concatenated along the channel dimension and further compressed by global average pooling to yield a tensor of shape [B, 384]. In the residual fusion stage, original embedding features were compressed via global average pooling to form a 128-dimensional vector, which was then projected to 384 dimensions through a linear layer to serve as the residual component. This residual feature was element-wise added to multi-scale convolutional features, followed by layer normalization and dropout regularization (dropout rate = 0.3). The refined features were finally mapped to 128 dimensions via a linear projection, generating the fixed-length sequence-level feature representation $F_{seq}\in R^{128}$.

#### 3.3.2. Structural Feature Extraction Module

The input to the structural feature extraction module consisted of four components: (1) adjacency matrices of peptides and proteins; (2) node features of shape [N, D], where N was the number of residues in the protein or peptide and D was the dimensionality of node features; (3) edge features with shape [E, 4], where E was the number of edges in the graph; and (4) coordinates of nodes constituting the graph.

Within the structural feature extraction module, we constructed a three-layer residual EGNN to capture structural features of proteins and peptides. Specifically, 1280-dimensional node features were first projected into a 256-dimensional hidden space. The structural features were then extracted by a three-layer residual EGNN, where each layer maintained a node count of 256. Within each layer, residual connections were established between input and output features, followed by layer normalization. Finally, 256-dimensional features output by a three-layer residual EGNN were passed through global max pooling and a linear transformation to obtain 128-dimensional structural features of peptides and proteins.

#### 3.3.3. Feature Fusion Module

To balance the importance of sequence and structural features, we constructed a feature fusion module adopting a “fusion within, then fusion between” strategy. Specifically, internal fusion referred to the integration of sequence and structural features within peptides and proteins, respectively. We employed an attention-based dual-modal fusion strategy, where the extracted 128-dimensional sequence features and 128-dimensional structural features were concatenated along the channel dimension to form a 256-dimensional feature vector. This vector was then fed into a linear mapping layer to generate a 2-dimensional weight vector, which was normalized using the Softmax function to obtain the attention weights $\alpha =\left[\alpha_{1},\alpha_{2}\right]$, with $\alpha_{1}+\alpha_{2}=1$. Here $\alpha_{1}$ and $\alpha_{2}$ denoted the relative importance of sequence features and structural features, respectively. Subsequently, the weighted sequence and structural features were concatenated to produce a 256-dimensional fused feature. External fusion, on the other hand, referred to the integration between peptides and proteins: 256-dimensional fused features of peptide and protein were concatenated into a 512-dimensional feature vector, which served as a comprehensive representation of their interaction.

#### 3.3.4. Peptide-Protein Interaction Prediction Module

The extracted comprehensive representation for PepPI prediction was utilized for binary classification through an MLP incorporated with skip connections. This network consisted of one input layer, four hidden layers, and one output layer. The input layer contained 512 nodes, while four hidden layers had 1024, 512, 256, and 128 nodes, respectively. Each hidden layer employed ReLU followed by batch normalization, with dropout rates set to 0.3, 0.3, 0.2, and 0.1, respectively. The output layer had one node.

Skip connections were introduced to alleviate gradient-related issues in deep neural networks. Specifically, 1024-dimensional features output by the first hidden layer were mapped to 256 dimensions and then added to 256-dimensional features from the third hidden layer, together serving as input to the fourth hidden layer. Finally, the threshold for determining the presence of PepPI was set to 0.5: a predicted value greater than 0.5 indicated an interaction; otherwise, no interaction.

### 3.4. Model Training

Model training adopted five-fold cross-validation, with 100 epochs per fold. The AdamW optimizer was employed for parameter updates, with momentum parameters $\beta_{1}=0.9$ and $\beta_{2}=0.999$, and a weight decay coefficient of 0.01. To mitigate GPU memory constraints, a gradient accumulation strategy was applied, where gradients were updated every 4 batches along with gradient clipping (with a threshold of 1.0) to prevent gradient explosion.

The initial learning rate was set to 0.001, and the learning rate scheduling combined warm-up with a cosine annealing restart strategy. Specifically, a linear warm-up scheduler was used for the first 10 epochs to gradually increase the learning rate from a low value to the initial learning rate. Afterward, the scheduler switched to a cosine annealing restart strategy with an initial cycle length of 10 epochs, a cycle multiplication factor of 2, and a minimum learning rate of 1 × 10^−6^. This approach enabled the learning rate to periodically decay and restart following a cosine function during training, facilitating the model’s ability to escape the local optima.

In this study, binary cross-entropy loss (Formula (1)) was used as the optimization objective:

(1) $$L=-\frac{1}{N}\sum_{i=1}^{N}\left[y^{\left(i\right)}\log\left({\hat{y}}^{\left(i\right)}\right)+(1-y^{\left(i\right)})\log\left(1-{\hat{y}}^{\left(i\right)}\right)\right]$$

where $y^{\left(i\right)}\in \left\{0,1\right\}$ denoted the true label of the sample i (1 for interacting pairs, 0 for non-interacting pairs); ${\hat{y}}^{\left(i\right)}\in \left[0,1\right]$ was the predicted probability output by the model after the sigmoid function; and N was the batch size.

### 3.5. Evaluation Metrics

In this study, PepPI prediction was formulated as a binary classification task. To comprehensively evaluate the classification performance of PepPI-DRN, we employed six evaluation metrics: accuracy (ACC), recall, specificity (Spec), F1 score (F1), area under the ROC curve (AUC), and area under the PRC curve (AUPRC).

$$ACC=\frac{TP+TN}{TP+FN+FP+TN}$$

$$Recall=\frac{TP}{TP+FN}$$

$$Spec=\frac{TN}{TN+FP}$$

$$F1=\frac{2\times Prec\times Recall}{Prec+Recall}$$

Here, true positive (TP) denotes a correctly predicted positive sample. True negative (TN) refers to a correctly predicted negative sample. False positive (FP) indicates a sample incorrectly predicted as positive, and false negative (FN) means a sample incorrectly predicted as negative. AUC represents the area under the ROC curve, where the *x*-axis corresponds to the false positive rate (FPR) and the *y*-axis to the true positive rate (TPR). AUPRC represents the area under the PRC curve, with the *x*-axis as recall and the *y*-axis as precision.

## 4. Conclusions

Computer-assisted screening of lead peptides from the venom gland transcriptome of *O. washanensis* has rarely been reported to date. Traditional computational methods struggle to meet the practical requirements of pharmacognosists in terms of both efficiency and prediction accuracy. To explore the pharmaceutical potential of peptides in the venom gland transcriptome of *O. washanensis*, this study developed PepPI-DRN, a deep learning model for candidate peptide screening based on deep residual networks. By integrating dual-modal information of sequences and structures of peptides and proteins, this model efficiently screened candidate peptides with potential interactions from the venom gland transcriptome of *O. washanensis* based on reported target proteins. Candidate peptides and proteins were further subjected to blind docking to form complexes. Results showed that some candidate peptides not only exhibited reasonable binding conformations with proteins but also possessed strong docking scores. Meanwhile, molecular dynamics simulations were performed to evaluate the stability of candidate complexes, confirming that some candidate peptide–target complexes displayed favorable structural stability. Although multiple experimental results showed that PepPI-DRN could preliminarily screen lead peptides interacting with given proteins from the venom gland transcriptome of *O. washanensis* with molecular docking and molecular dynamics simulation, the model and this study still had several limitations:

(1) In addition to peptide sequence information, PepPI-DRN also depends on peptide structural information. However, since the venom gland transcriptome of *O. washanensis* currently provides only peptide sequence data, protein tertiary structure prediction tools such as ColabFold and AlphaFold must be employed to predict the peptide structures.

(2) The venom gland transcriptome of *O. washanensis* contains over 300,000 peptide sequences. However, as a preliminary exploratory study of lead peptides, this work randomly selected and analyzed only 30,000 of these peptide sequences without systematic screening of the entire transcriptome. The results are therefore incomplete to some extent.

(3) In the benchmark dataset, negative samples were generated by randomly pairing peptides from positive samples with targets after removing duplicates of positive samples. Although some studies adopted the same strategy for negative sample construction, a small fraction of these negative samples may still exhibit interactions between peptides and targets, thereby introducing noise into model training and compromising model performance.

(4) The benchmark dataset used is limited in size, resulting in insufficient and incomplete representation learning of peptide and protein sequences and structures by PepPI-DRN. In addition, there is also a lack of wet-lab experimental validation to confirm whether lead peptides exhibit activity against targets.

In future work, we will systematically expand the PepPI dataset and further improve the benchmark dataset. Meanwhile, we will construct a deep learning model based on pure sequence representation. Guided by the key pathogenic targets, we aim to achieve full-library screening of lead peptides from the venom gland transcriptome of *O. washanensis*, laying a theoretical and methodological foundation for the development of novel peptide drugs derived from spiders.

## Figures and Tables

**Table 1:** Performance comparison of PepPI-DRN against four state-of-the-art methods on an independent test set.

Model	ACC	Recall	Spec	F1	AUC	AUPRC
PIPR	0.755	0.785	0.771	0.762	0.816	0.785
SCNN	0.729	0.745	0.737	0.734	0.790	0.753
TAGPPI	0.774	0.824	0.724	0.785	0.844	0.819
TPepPro	0.855	0.884	0.827	0.859	0.922	0.909
PepPI-DRN	0.884	0.964	0.804	0.893	0.911	0.880
*Impr.* (%)	3.4↑	9.0↑	2.8↓	4.0↑	1.2↓	3.2↓

Note: *Impr*. (%) indicates the percentage improvement achieved by PepPI-DRN over the best-performing model among all compared methods. Symbol ↑ denotes performance improvement, while ↓ denotes performance degradation.

**Table 2:** 95% confidence interval and interval width of each evaluation metric.

Merics	Point Estimation	95% CI	CI Width
ACC	0.8839	[0.8214, 0.9375]	0.1161
Recall	0.9643	[0.9107, 1.0000]	0.0893
Spec	0.8036	[0.6909, 0.8980]	0.2071
F1	0.8926	[0.8276, 0.9440]	0.1164
AUC	0.9131	[0.8489, 0.9646]	0.1157
AUPRC	0.8833	[0.7797, 0.9631]	0.1834
Precision	0.8308	[0.7344, 0.9105]	0.1761
MCC	0.7780	[0.6659, 0.8787]	0.2128

**Table 3:** Performance comparison of residual equivariant graph neural network with different numbers of layers.

Number of Layers	ACC	Recall	Spec	F1	AUC	AUPRC
2	0.7332	0.811	0.6546	0.7495	0.8196	0.8113
3	0.8642	0.8827	0.8436	0.8665	0.9308	0.9199
4	0.8335	0.8553	0.8175	0.8161	0.8638	0.8971
5	0.8389	0.8819	0.7992	0.8438	0.9082	0.8913

**Table 4:** Top-10 candidate peptide-protein pairs with highest predicted interaction probabilities based on PepPI-DRN.

Rank	Peptide	Protein	Probability	Rank	Peptide	Protein	Probability
1	DN131049_c0_g1	T779	0.9654	6	DN50683_c0_g1	T779	0.9642
2	DN87155_c0_g1	T779	0.9653	7	DN166034_c0_g1	T779	0.9639
3	DN23957_c1_g2	T779	0.9647	8	DN166034_c0_g1	T675	0.9639
4	DN79336_c0_g1	T675	0.9645	9	DN79336_c0_g1	T779	0.9637
5	DN100998_c0_g1	T779	0.9644	10	DN146228_c0_g1	T675	0.9631

## Data Availability

The original data presented in the study are openly available in PepPI-DRN at http://github.com/dldxzx/PepPI-DRN (accessed on 15 August 2026).
